# A dual-purpose humanized mouse model for testing antiviral strategies against both SIV and HIV

**DOI:** 10.3389/fimmu.2024.1491481

**Published:** 2024-11-04

**Authors:** Ella Barnett, Snehal Kaginkar, Kimberly Schmitt, Leila Remling-Mulder, Ramesh Akkina

**Affiliations:** Department of Microbiology, Immunology, and Pathology, Colorado State University, Fort Collins, CO, United States

**Keywords:** a dual-purpose hu-mouse model for HIV and SIV drug testing, anti-retroviral drug evaluation against SIV and HIV in humanized mice, a small animal model for SIV, emtricitabine/elvitegravir/tenofovir disoproxil fumarate (FTC/EVG/TDF) treatment for SIVmac239, emtricitabine/bictegravir/tenofovir alafenamide fumarate (FTC/BIC/TAF) treatment for SIVmac239, cART evaluations on SIV and HIV in humanized mice, NHPs and humanized mouse models for antiviral drug testing, humanized mice for HIV cure studies

## Abstract

Nonhuman primate (NHP) models employing simian/simian-human immunodeficiency viruses (SIV/SHIVs) played a major role in the study of HIV pathogenesis, latency, and cure studies in a preclinical setting. However, it took many years to arrive at the current effective triple drug ARV regimen against SIV due to the genetic differences with that of HIVs. Since new combinations of drugs will be used in the evolving HIV cure studies, a small animal model would be ideal to determine their efficacy against the commonly used SIVs such as SIVmac239 to triage ineffective drugs prior to their application in NHPs. We recently determined that humanized mice (hu-mice) with a transplanted human immune system are permissive to SIVmac strains in addition to HIVs. Based on this novel finding, here we evaluated the utility of this dual-purpose hu-mouse model to test different ART regimens against SIVmac239. Infected mice showing chronic viremia were treated with a combination anti-retroviral treatment (cART) regimen consisting of emtricitabine/elvitegravir/tenofovir disoproxil fumarate (FTC/EVG/TDF). Full viral suppression was seen for several weeks in SIVmac239-infected and treated mice similar to that seen with HIV-1 BaL virus used as a control. However, viral rebound was eventually observed in SIVmac239 infected mice during the treatment period, suggesting viral escape compared to HIV-1 BaL with which viral suppression was fully sustained. Next, a cART regimen consisting of emtricitabine/bictegravir/tenofovir alafenamide fumarate (FTC/BIC/TAF) was similarly evaluated. Our results showed that this ARV regimen was fully effective in rapidly suppressing both SIVmac239 and HIV-1 BaL. Complete viral suppression was maintained until treatment interruption after which viral loads rebounded. These findings highlight the utility of humanized mice for *in vivo* screening of new combinations of ARV compounds against various SIVs prior to employing them in NHPs. In addition to identifying new effective cART regimens against SIVs, this model would also be amenable to evaluating immunotherapeutic strategies using broadly neutralizing antibodies, LRAs and novel therapeutics in comparative cure studies of SIV and HIV.

## 1 Introduction

Animal models play an important role in drug discovery and evaluation. Humanized mice (hu-mice) have been very helpful in many HIV research areas such as viral transmission, pathogenesis, therapies and prevention as well as in employing novel strategies such as gene therapy (1–8). Hu-HSC mice are prepared by engrafting human blood forming CD34^+^ stem cells from sources such as cord-blood into neonatal mice. In contrast, BLT mice are prepared by transplantation of human fetal liver and thymic tissues plus injection of autologous hematopoietic stem cells (HSC). Hu-mice harbor a full complement of human T, B and NK cells, as well as macrophages and dendritic cells. Hu-mice are highly susceptible to HIV-1 infection and permit the study of host responses (1–4, 9). While the majority of hu-mouse studies focused on HIV itself as the agent of investigation, we also employed these mice as a human surrogate model to better understand the origin and evolution of HIVs from their progenitor ancestral SIV viruses native to chimpanzees and sooty mangabeys (10–12). It was found that the SIVcpz (ancestor for HIV-1) (12–14) and SIVsm (ancestor for HIV-2) (11) can cause productive infection and chronic viremia in hu-mice. Serial passages of these viruses spanning many months in hu-mice led to evolutionary genetic changes towards HIVs. Many non-synonymous mutations seen in different regions of the genomes became fixed in later passages demonstrating human adaptive changes (11, 12, 15). During these studies, we also became interested in developing novel animal models to study SIVs *in vivo*. In this context, we made a novel observation that hu-mice are also permissive to infection with SIVmac strains. Hu-mice challenged with SIVmac239 became readily infected and showed persistent viremia (15). After further passaging the virus in hu-mice, helper CD4 T cell decline was seen during the chronic phase of infection akin to that seen in the NHP system

HIV related research commonly employs SIVmac239 virus since infected rhesus macaques display many of the same pathologies seen in human AIDS patients (16–18). However, HIV-1 and SIVmac viruses differ significantly. SIVmac239 belongs to the SIVsm/HIV-2 lineage whereas HIV-1 belongs to the SIVcpz lineage (19, 20). In the context of antiretroviral drugs, not all compounds effective against HIV show comparable efficacy on SIV due to differences in their genetic makeup. For example, anti-HIV-1 non-nucleoside reverse transcriptase inhibitors (NNRTIs) have little to no effect on HIV-2 and SIV infections (21–23). For HIV, there is ongoing development of new drugs which are expected to used in different combinations (24–26). As new generation drugs are expected to be used in HIV cure studies involving SIVs, a small animal model would be ideal to determine their efficacy against the commonly used SIVs such as SIVmac239 to triage ineffective drugs prior to their application in NHPs. An animal model that permits testing of drugs for both HIV and SIV will have many advantages, such as its application in ART comparative studies. In addition, preliminary data gained on the efficacy of new antiretrovirals from *in vivo* mouse experiments will help to better inform experiments using rhesus macaques and to conserve precious resources. With this as a background, here we evaluated hu-mice as a hybrid dual-use model for testing therapeutics against both HIV and SIV infections. Our results showed that the hu-mouse animal model can help bridge the testing capability for both anti-HIV and anti-SIV drugs in comparative studies.

## 2 Materials and methods

### 2.1 Preparation of hu-HSC mice

Humanized BALB/c/RAG1 or RAG2-/-gc-/- mice (hu-HSC mice, also referred to as hu-mice) were prepared by injecting human CD34 hematopoietic progenitor cells intra-hepatically into newborn mice as we described previously (27). Mice were maintained at the Colorado State University Painter Animal Center. These studies have been reviewed and approved by the CSU Institutional Animal Care and Use Committee. Briefly, human fetal liver derived CD34 cells were cultured for 24 hours in cytokine media (1, 5). To prepare hu-mice, neonatal (1-3 day old) mouse pups were sub-lethally irradiated (350 rads) prior to intrahepatic injection of 0.5-1x10^6^ CD34+ cells (11, 27, 28). Engrafted mice were screened for human cell engraftment at 10-12 week post-reconstitution. Peripheral blood was collected, and the red blood cells were lysed using the Whole Blood Erythrocyte Lysing Kit (R&D Systems, Minneapolis, MN). White blood cells were stained with fluorophore conjugated antibodies to human hCD45-APC, hCD3-FITC, and hCD4-PE (BD Pharmingen, San Jose, CA) and FACS analyzed to confirm human cell engraftment (27). The human cell engraftment levels varied with an average of 58%. The presence of chronic viremia in infected mice was confirmed prior to ART commencement.

### 2.2 SIVmac239 viral stock preparation and infection of hu-mice

The SIVmac239 full length infectious clone was transfected into HEK293T cells to generate viral stocks. After 48 h, the viral supernatant was collected, filtered and stored at -80°C. Thawed aliquots of this viral supernatant was utilized for the *in vivo* infections. The SIVmac239 viral titer was determined in TZM-bl reporter cells (12, 29, 30). Cohorts of hu-HSC mice with high human hematopoietic engraftment levels were inoculated with ~200 µL of virus suspension (10^5.5^ TCID50) via intraperitoneal (i/p) injection with one of the respective viruses (SIVmac239 or HIV-1 BaL). Viral infection was monitored by qRT-PCR from plasma collected weekly. General aspects of SIVmac239 infection dynamics and immune cell profiles in hu-mice were described in our previous studies (15).

### 2.3 Plasma viral load determination by qRT-PCR

Peripheral blood was collected from the tail vein of the mice on a weekly basis to assess plasma viral loads (PVL). Viral RNA was extracted from plasma with the E.Z.N.A. Viral RNA kit (OMEGA bio-tek, Norcross, GA) and was then used to determine viral loads via qRT-PCR. Virus-specific primers and probes used for the qRT-PCR reactions were designed against the *ltr* regions of SIVmac239 (GenBank accession number: M33262.1) and HIV-1 BaL and are listed as follows: 1.) SIVmac239: forward (5’-GCAGGTAAGTGCAACACAAA-3’), reverse (5’-CCTGACAAGACGGAGTTTCT-3’) and probe (5’-FAM/AAGATAGAG/ZEN/TGGGAGATG-3’), and 2.) HIV-1 BaL: forward (5’- GCCTCAATAAAGCTTGCCTTGA-3’), reverse (5’- GGCGCCACTGCTAGAGATTTT-3’) and probe (5’-FAM/AAGTAGTGT/ZEN/GTGCCCGTCTGTTRTKTGACT/3lABkFQ-3’) (12). A qRT-PCR reaction was performed using these primer/probe sequences with the with the iTaq Universal Probes One-Step Kit (Bio Rad Labs, Hecules, CA) and the following cycling conditions: 50°C for 10 min, 95°C for 2 min, followed by 40 cycles of 95°C for 15 sec and 54°C (SIVmac239) or 64°C (HIV-1 BaL) for 30 sec in the Bio Rad C1000 Thermo Cycler with the CFX96 Real-Time System (Bio Rad, Hercules, CA).The limit of detection for both SIVmac239 and HIV-1 BaL was 1,000 viral RNA copies/mL.

### 2.4 Drug dosing and treatment

**Figure 1:**
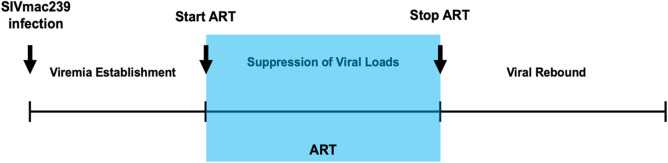
Schematic of viral infection, ARV treatment and treatment interruption. Hu-mice were infected with either SIVmac239 or HIV-1 BaL by i/p route. Post infection and after establishment of chronic viremia, ARV treatment was commenced and continued for 12-16 weeks (shaded area) after which the treatment was withdrawn. Viral loads from plasma were determined on a weekly basis.

To evaluate the utility of hu-mouse model for anti-viral drug testing against SIVmac, the following anti-retroviral drugs were used in different combinations and doses (24–26). The non-nucleoside RT inhibitor emitricitabine (FTC), and tenofovir disoproxil fumarate (TDF) and tenofovir alafenamide fumarate (TAF) which are different formulation of the RT inhibitor tenofovir (TFV). Two versions of integrase strand transfer inhibitors (INSTI), namely elvitegravir (EVG) and bictegravir (BIC) were also used. The two treatment regimens were composed of three drug combinations, FTC/TDF/EVG (200/200/400mg per 60kg human) and FTC/TAF/BIC (200/25/50mg per 60kg human). The equivalent dose for a 20-gram mouse was calculated from the human equivalent dose (HEC) by the allometric dosing scale which takes into account body weight and basal metabolic rate (31). Here we used twice the HEC for drug administration. The dose for FTC/TDF/EVG combination was calculated as 1.65mg FTC, 1.65mg TDF, and 3.29mg EVG per mouse/day. Mice treated with a second combination of FTC/TAF/BIC received doses of 1.65mg FTC, 0.21mg TAF, and 0.40mg BIC per mouse/day via food. The three drug cocktails were incorporated into Dietgel Boost cup (ClearH20, Wesbook, Maine).Groups of SIVmac239 and HIV-1 BaL (5 for each group) were either treated or untreated. Viral loads were assayed by RT-PCR to assess the drug efficacy. The schematic of treatment and its cessation are depicted in **Figure 1**.

## 3 Results

### 3.1 Anti-retroviral drug efficacy on SIVmac239 *in vivo* can be evaluated in humanized mice

**Figure 2:**
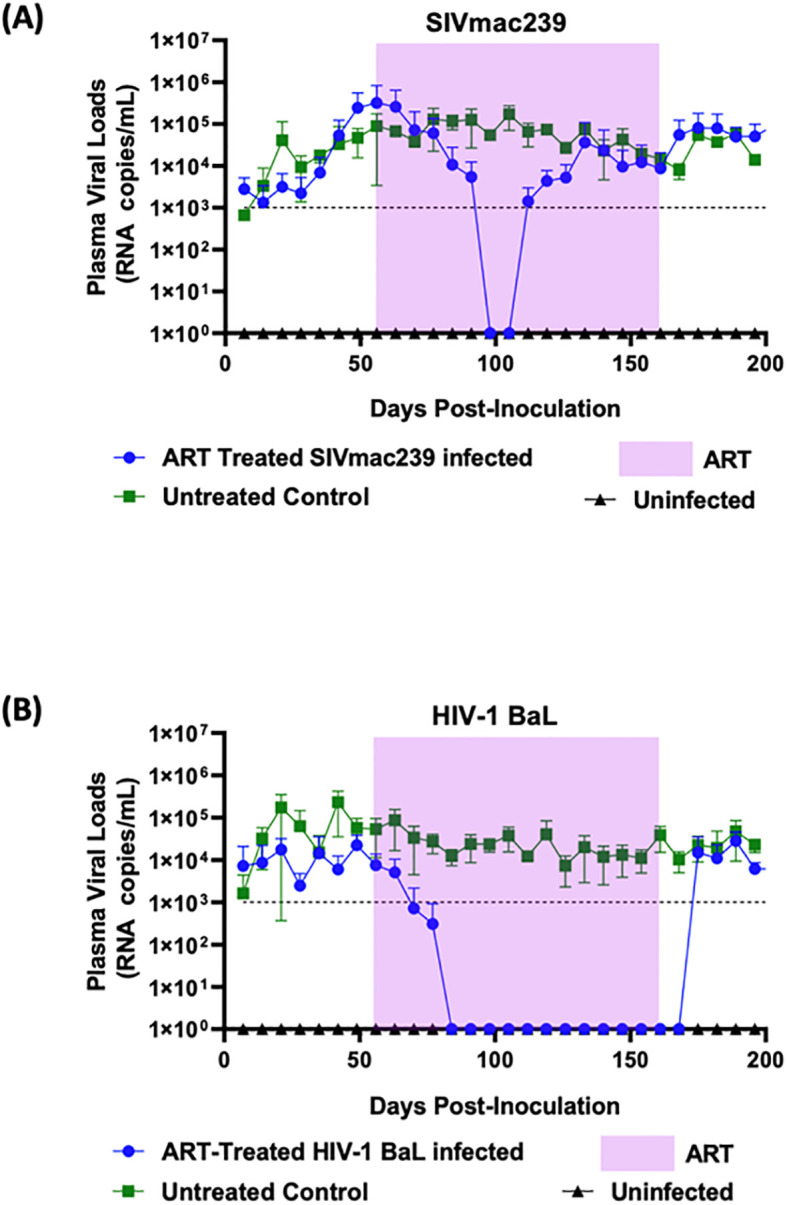
Combinatorial ARV treatment with FTC/EVG/TDF suppresses SIVmac239 infection transiently in hu-mice: Hu-mice were infected with either **(A)** SIVmac239 or **(B)** HIV-1 BaL by i/p route. Post-infection, after establishment of chronic viremia, ARV treatment was commenced with the three-drug combination FTC/EVG/TDF in a dietary supplement. Treatment was continued for 16 weeks (shaded area) before ATI. Plasma viral loads were measured weekly. Infected and untreated as well as uninfected mice were used as controls.

To develop the hu-mouse model as a pre-screen to test ARV compounds on SIVmac239, we evaluated two different ARV combinations, both containing three drugs. Drug dosages were calculated using allometric scaling. The same drug combinations were used for treatment of both SIVmac239 and HIV-1 BaL infected hu-mice for comparison. Infected mice were allowed to develop chronic and persistent viremia prior to commencing antiviral treatments. As can be seen in **Figure 2**, steady state viral loads were established by seven weeks post-infection and maintained thereafter. FTC/EVG/TDF drug treatment commenced eight weeks post-infection and continued for sixteen weeks followed by cessation of therapy. In HIV-1 BaL infected mice given FTC/EVG/TDF, there was a rapid decline in viral loads below the baseline of viral detection within two weeks of treatment (**Figure 2B**). This trend continued until cessation of therapy. HIV-1 BaL viral rebound was observed within two weeks post-treatment cessation and viral loads returned to pre-treatment levels thereafter. Viral suppression was also achieved in SIVmac239 infected and FTC/EVG/TDF treated mice. As shown in **Figure 2A**, the rate of SIVmac239 viral load decline was slower compared to that seen in HIV-1 BaL infected mice, taking six weeks versus two weeks. This data shows that hu-mice can be used as a NHP surrogate model to evaluate anti-retroviral drugs *in vivo*. However, viral rebound was noted in SIVmac239 infected mice after two weeks of viral suppression despite the continuation of drug treatment, indicating development of drug resistance against this regimen. SIVmac239 viral loads continued to rise and eventually reached comparable levels to that of untreated mice. In contrast, with HIV-1, the same drug regimen showed sustained antiviral effect until drug interruption.

### 3.2 Complete SIVmac239 anti-viral suppression was achieved with the FTC/BIC/TAF combination

**Figure 3:**
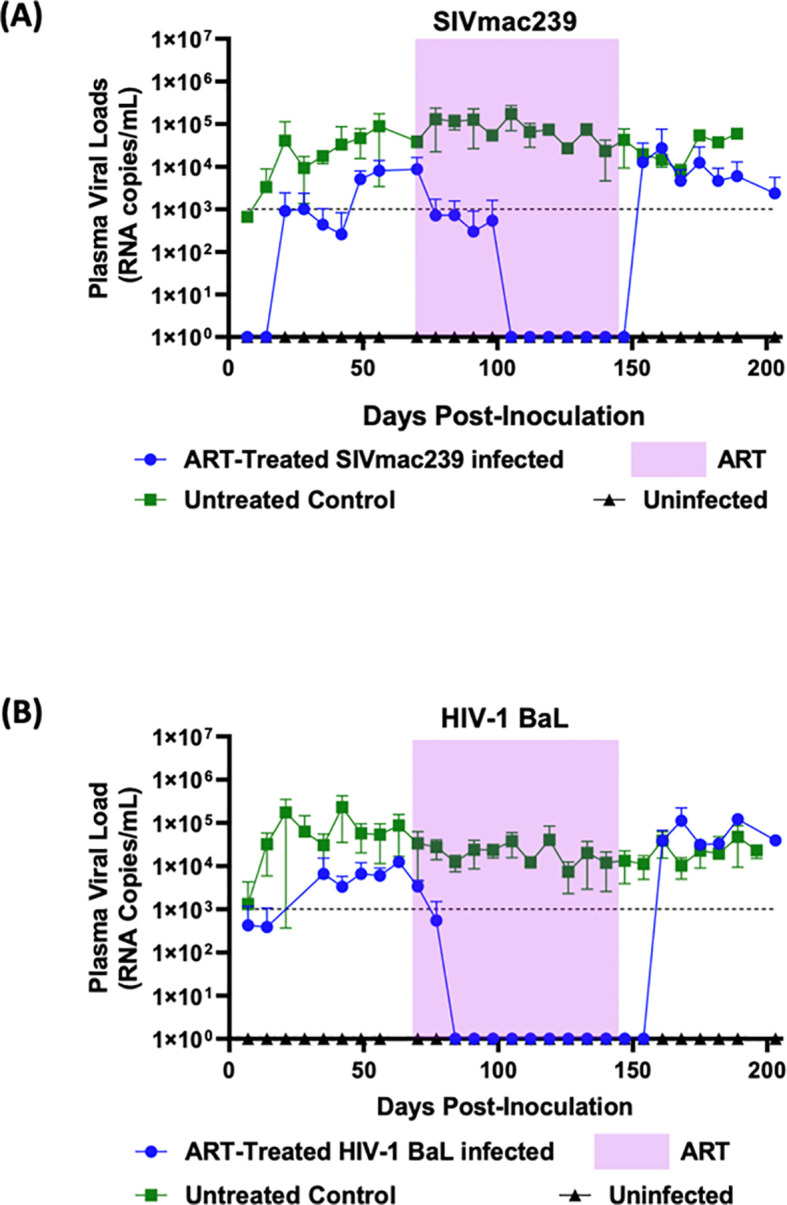
Combinatorial ARV treatment with FTC/BIC/TAF fully suppresses SIVmac239 infection in hu-mice: Hu-mice were infected with either **(A)** SIVmac239 or **(B)** HIV-1 BaL by i/p route. Post-infection, after establishment of chronic viremia, ARV treatment commenced with the three drug combination FTC/BIC/TAF in a dietary supplement. Treatment was continued for twelve weeks (shaded area) before ATI. Plasma viral loads were measured weekly. Infected and untreated as well as uninfected mice were used as controls.

Based on the results from above, we proceeded to test a different combination of drugs consisting of FTC/BIC/TAF on hu-mice infected with SIVmac239 and HIV-1 BaL (**Figure 3**). The twelve-week FTC/BIC/TAF treatment commenced seven weeks post-infection. In infected mice prior to treatment, viral loads reached a plateau indicating viral persistence and a steady state of chronic viremia. Once HIV-1 BaL infected mice began ART, rapid viral load decline was noticed, reaching undetectable levels within two weeks (**Figure 3B**). This viral suppression trend continued until after cessation of drug administration. Viral rebound occurred after two weeks post-ART cessation, with viral load levels reaching comparable levels to those of infected and untreated mice. Regarding SIVmac239 infected and treated mice, complete viral suppression was also observed but the rate of viral decline was slower than that seen in HIV-1 BaL infected and treated mice, taking five weeks (**Figure 3A**). In contrast to FTC/EVG/TDF treatment, the FTC/BIC/TAF drug combination was fully effective in suppressing viral loads until after cessation of therapy, indicating full efficacy with this combination. SIVmac239 viral rebound could be seen within a week after treatment interruption with viral loads returning to pre-treatment levels.

## 4 Discussion

To our knowledge, this is the first report wherein SIV and HIV therapeutics were evaluated in comparative studies using the same small animal model, namely, humanized mice. Thus far nearly every class of ART has been tested in NHPs (18–22). For example, initially shown to be effective in SIV PrEP studies, the NRTI drug tenofovir (TFV), and now it’s derivatives with improved PKs and lower toxicities continue to be widely employed. It took more than a decade to identify a combinatorial ART regimen with three ARV drugs to attain durable viral suppression of SIV in NHPs (32). A common cART regimen used today for SIV consists of tenofovir disoproxil fumarate (TDF), emtricitabine (FTC), and dolutegravir (DTG) (32–35). While effective, it is important that new generations of anti-HIV drugs be used in SIV research to better emulate the constantly evolving HIV-1 treatment regimens in the quest for identifying the ultimate cure strategies using the SIV model. As mentioned above, in our previous studies we found that the hu-mouse model is susceptible to infection with a variety of SIVs including the most commonly used SIV strain, SIVmac239 (11, 12, 15). This formed the basis for evaluating the potential of the hu-mouse model as a novel dual-purpose small animal model in which both HIVs and SIVs can be evaluated for comparative purposes involving an array of therapeutic compounds and biologics such as broadly neutralizing antibodies to determine efficacies.

As expected, in the HIV-1 BaL infected mice used as controls, cART employing TDF/FTC/EVG showed full viral suppression within two weeks after commencing the treatment. Viral loads remained undetectable throughout the treatment period and a rapid viral rebound ensued upon treatment interruption (**Figure 2B**). Similar to that with HIV-1, SIVmac239 infected and treated mice also displayed viral suppression. However, this was achieved six weeks into the treatment period versus two weeks with HIV-1 (**Figure 2A**). Also, compared to HIV-1, full viral suppression of SIVmac239 lasted for only two weeks before viral rebound during the treatment period, suggesting development of drug resistance. Thus, these findings, in addition to determining the drug efficacy, also showed the utility of hu-mice in assessing treatment failure during prolonged therapy with a specific drug regimen. Further analysis of results showed that animals with higher viral loads prior to drug treatment were relatively more refractive to suppression by the ART regimen compared to mice with lower initial plasma viral loads (data not shown). To achieve a potentially more sustained suppression of SIVmac239, we next assessed a different combination of drugs consisting of FTC/BIC/TAF. Tenofovir alafenamide (TAF) is the prodrug form of TFV with a greater antiviral potency and a better renal safety profile. With this regimen, SIVmac239 infected and treated mice showed a complete and sustained viral suppression (**Figure 3A**). As expected, HIV-1 infected mice also showed full viral suppression. (**Figure 3B**). However, with SIVmac239, viral load decline was slower, taking three weeks longer than HIV-1 infected mice. As can be seen, in contrast to to the FTC/EVG/TDF cART, the FTC/BIC/TAF drug combination proved to be fully effective in SIVmac239 viral suppression. Viral rebound with SIVmac239 following treatment cessation indicates that SIVmac239 establishes viral latency in this hu-mouse model, similar to HIV-1.

Collectively, the above data showed that the hu-mouse model can be exploited to evaluate antiviral compounds against SIV in an *in vivo* system thus setting the stage for rapidly assessing new anti-HIV compounds currently in the pipeline. Different SIVs in addition to SIVmac239 such as various SHIVs with different genetic composition can also be tested for treatment efficacy with different compounds. Thus, comparative data on the effectiveness of various drugs on these different viruses can be derived quickly for later application. Many aspects of SIV pathogenesis, viral persistence, and latency can be also studied. Hu-mice will provide direct drug efficacy comparisons with reduced costs and in less time thus help accelerate the testing of larger numbers of promising antiretroviral compounds and antibodies. This dual-purpose hu-mouse model breaks new ground on other fronts as well. Evaluation of different classes of LRAs on both viruses using the same system will help discern any inherent differences. It is now known that there are subtle differences between SIV and HIV in establishing latency, the proportion of defective viruses generated, and preferred integration sites in the genome. Such differences can be studied in hu-mice to gain knowledge on the comparative aspects in this important area. Another area of work that will potentially benefit from this model is establishment of SIV sexual transmission by mucosal routes such as the vaginal route. This would be of high interest to pursue to further increase the breadth of this model for more novel experiments in the future. It can be foreseen that this dual-purpose hu-mouse model will benefit both the HIV and SIV research groups fostering further innovation in cure studies due to its versatility and simplicity.

## Data Availability

The original contributions presented in the study are included in the article/supplementary material. Further inquiries can be directed to the corresponding author.
